# Varicella-Zoster as a Cause of Aseptic Meningitis in an Immunocompetent Young Patient With Skin Rash

**DOI:** 10.7759/cureus.8745

**Published:** 2020-06-21

**Authors:** Harith Alataby, Ragu Gautam, Michael Yuan, Jay Nfonoyim

**Affiliations:** 1 Internal Medicine, Richmond University Medical Center, Staten Island, USA; 2 Pulmonary and Critical Care, Richmond University Medical Center, Staten Island, USA

**Keywords:** varicella-zoster, immunocompetent patient, aseptic meningitis, skin rash

## Abstract

We present a case of aseptic meningitis due to Varicella-Zoster infection in an immunocompetent patient. Varicella-Zoster virus (VZV) causes chickenpox disease in children, teens, and young adults. Typically, it runs its course and stays dormant in nerve tissue, which can get reactivated in elderly, immunocompromised patients. Frequently, reactivation results in the painful dermatomal rash of herpes zoster, but in sporadic cases, it can cause meningitis or encephalitis in the immunocompromised population. Our case demonstrates a healthy immunocompetent adult male who presented with headache, fever, mild neck stiffness, and painless right-sided abdominal skin rash and was later diagnosed with VZV meningitis via polymerase chain reaction (PCR) of the cerebrospinal fluid (CSF). We are reporting this case due to its rarity, and the challenging nature of its diagnosis and treatment. In the hospital, he was treated with IV acyclovir for three days and discharged home on 14 days of oral valacyclovir. Our case demonstrates the importance of having a high degree of suspicion, even if the presentation is unexpected and atypical.

## Introduction

Varicella-Zoster virus (VZV) is the third virus in the human herpes virus family (HHV-3). It is an enveloped, double-stranded, linear virus. Varicella-zoster commonly causes two different infections: chickenpox and shingles. Chickenpox is typically found in children, specifically in those who are missing vaccinations [1]. It presents as a vesicular rash that begins on the trunk and then spreads to the face and extremities. Lesions in all stages of development are an essential indicator of chickenpox. Once an individual has recovered symptomatically from the chickenpox, the virus can remain dormant in the dorsal root or trigeminal ganglion permanently [1]. Later in life, if the infected individual is physiologically stressed to a high enough degree, the virus can re-emerge in the form of shingles, which typically presents as a vesicular rash that appears in a dermatomal pattern, not crossing the midline. It will eventually regress but can lead to postherpetic neuralgia and recurrence [2].

Other less common conditions caused by Varicella-Zoster include meningitis, herpes zoster ophthalmicus, vasculitis, encephalitis, pneumonia, and bronchitis. These frequently are found in immunocompromised patients, and much more rarely, in immunocompetent patients [3]. In an immunocompromised patient such as with HIV, the VZV will often reactivate in more than one dorsal root ganglion, quickly spreading to both the skin and the central nervous system (CNS). These patients may or may not present with the typical dermatomal rash [4].

If a patient is found to be immunocompromised, there is a high degree of clinical suspicion for varicella-zoster induced viral meningitis or encephalitis. However, this is not the case for immunocompetent patients, where the percentage of aseptic viral meningitis and encephalitis caused by Varicella-Zoster is often underestimated by clinicians [5].

## Case presentation

A 40-year-old male with no significant past medical history presented to the ED complaining of severe headaches for three days. The headache was insidious in onset, generalized, continuous, and throbbing in nature. No aggravating and relieving factors. The headache was associated with severe neck pain, photophobia, multiple episodes of vomiting (two to three times per day), diarrhea (three to four times per day), fever, and chills.

The patient stated that he had painless erythematous lesions on the right side of the abdomen at the level of the umbilicus which started seven days prior to the admission.

On general physical examination, the patient was alert and oriented to time, place, and person. The patient’s blood pressure was 120/80 mmHg, pulse rate was 80 beats per minute, respiratory rate was 18 cycles per minute, and the temperature was 98°F. Neurological examination showed the patient had neck stiffness and positive Kernig’s sign. The rest of the CNS exam was unremarkable. Skin examination showed a red rash with tiny, fluid-filled blisters. No abnormalities were detected on other systemic examinations.

Laboratory wise, complete blood count and metabolic panel were within normal ranges, and blood culture was negative. CT head without contrast was unremarkable for any intracranial abnormalities. Urgently, the patient underwent a lumbar puncture and cerebrospinal fluid (CSF) analysis, which showed an opening pressure of 28 mmHg, white blood cell count of 309 (99% lymphocytes and polymorphonucleocytes 1%), total protein of 139; Gram stain and culture were negative. CSF polymerase chain reaction (PCR) test was positive for the VZV. Additionally, skin lesion PCR was positive for VZV too.

Viral meningitis was confirmed, and the patient received IV fluids and acyclovir 1000 mg three times a day for four days. The following day the patient’s headache had improved significantly; by the third day, the patient’s signs and symptoms had resolved. The patient was monitored in the hospital for seven days and was discharged on 14 days course of oral valacyclovir 1000 mg three times a day. The patient was followed-up in three weeks of hospital discharge without any neurological complications.

## Discussion

Aseptic meningitis is a term referring to patients who have signs and symptoms of meningitis but are negative for routine bacterial cultures. Causes of aseptic meningitis are variable, including viruses, fungi, and even adverse drug reactions [6-7]. It is essential to distinguish between these differentials due to their individual treatment regimens [8]. Our case is an excellent example of varicella-zoster meningitis’s presentation. Not only it describes the presentation of a rare disorder, but it is also a reminder that it is crucial to maintain a high degree of clinical suspicion for varicella-zoster when treating aseptic meningitis. The immunization status of a patient is vital to consider, but should not be used to rule out varicella-zoster entirely. The viral causes are important to be noted because they all present with a similar result after a spinal tap. The CSF is typically clear, with normal range opening pressure, lymphocytic predominance, mildly raised protein, and low to normal glucose [9]. CSF PCR is the backbone of the diagnosis and differential between the types of viral meningitis.

Early starting of the antiviral is crucial and delayed or lack of treatment could have resulted in severe neurological complications or even patient mortality. In light of these severe consequences, IV acyclovir 10 mg/kg three times a day is recommended for VZV meningitis for 10-14 days [10]. Our patient has significantly improved by receiving IV acyclovir for four days; then the decision was made to switch him to oral valacyclovir 1000 mg twice a day to finish the total antiviral course of 14 days.

## Conclusions

The described case was an excellent example of VZV meningitis in an immunocompetent patient. Even young and healthy patients with clinical features of shingle can present with VZV meningitis. We highlight the importance of considering VZV as a possible cause for meningitis and recommend early diagnostic lumbar puncture with detailed CSF analysis, including PCR, to identify VZV. Early initiation of treatment with IV acyclovir with or without switching to oral valacyclovir often has an excellent clinical outcome.

## References

[1] Cohen JI (2008). Varicella-zoster virus: general features. Encyclopedia of Virology (Third Edition).

[2] Arvin AM (1996). Varicella-zoster virus. Clin Microbiol Rev.

[3] Chiang F, Panyaping T, Tedesqui G, Sossa D, Costa Leite C, Castillo M (2014). Varicella zoster CNS vascular complications: a report of four cases and literature review. Neuroradiol J.

[4] Wiegering V, Schick J, Beer M (2011). Varicella-zoster virus infections in immunocompromised patients - a single centre 6-years analysis. BMC Pediatrics.

[5] Tavazzi E, Minoli L, Ferrante P (2008). Varicella zoster virus meningo-encephalo-myelitis in an immunocompetent patient. Neurol Sci.

[6] Frantzidou F, Kamaria F, Dumaidi K, Skoura L, Antoniadis A, Papa A (2008). Aseptic meningitis and encephalitis because of herpesviruses and enteroviruses in an immunocompetent adult population. Eur J Neurol.

[7] Lee BE, Davies HD (2007). Aseptic meningitis. Curr Opin Infect Dis.

[8] Jarrin I, Sellier P, Lopes A (2016). Etiologies and management of aseptic meningitis in patients admitted to an internal medicine department. Medicine (Baltimore).

[9] Ihekwaba UK, Kudesia G, McKendrick MW (2008). Clinical features of viral meningitis in adults: significant differences in cerebrospinal fluid findings among herpes simplex virus, varicella zoster virus, and enterovirus infections. Clin Infect J.

[10] Kaewpoowat Q, Salazar L, Aguilera E, Wootton SH, Hasbun R (2016). Herpes simplex and varicella zoster CNS infections: clinical presentations, treatments and outcomes. Infection.

